# 1146. Cost-effectiveness of the Pentavalent Meningococcal Vaccine (MenABCWY): A steady-state cohort modelling approach

**DOI:** 10.1093/ofid/ofad500.987

**Published:** 2023-11-27

**Authors:** Katharina Schley, Jessica V Presa, Vincenza Snow, Alejandro D Cane, Paula Peyrani, Ray Farkouh, Shannon M Sullivan, Hossein Zivaripiran, Diana Teloian, Jörgen Möller, J Jaime Caro

**Affiliations:** Pfizer Pharma GmbH, Berlin, Berlin, Germany; Pfizer Inc., Collegeville, Pennsylvania; Pfizer Vaccines, Collegeville, PA; Pfizer, Collegeville, Pennsylvania; Pfizer, Inc, Collegeville, Pennsylvania; Pfizer, Inc., New York, New York; Evidera Inc., Ivry-sur-Seine Cedex, Ile-de- France, France; Evidera Inc., Ivry-sur-Seine Cedex, Ile-de- France, France; Evidera Inc., Ivry-sur-Seine Cedex, Ile-de- France, France; Evidera Inc, Lund, Skane Lan, Sweden; Evidera Inc., Ivry-sur-Seine Cedex, Ile-de- France, France

## Abstract

**Background:**

Invasive meningococcal disease (IMD) is associated with a high case fatality rate and serious long-term sequelae (LTS). The current US vaccination platform targets serogroups ACWY with a routine recommendation in adolescents (11-12 years, with a booster at 16 years) and serogroup B (16-23 years) based on shared clinical decision making. A novel pentavalent meningococcal vaccine (MenABCWY) may improve protection against all five serogroups and simplify vaccination schedule. The study objective was to assess the cost-effectiveness of MenABCWY from a societal perspective in the US.

**Methods:**

A steady-state model was developed to estimate the impact of various vaccination strategies on IMD cases, LTS, and deaths. Cohorts of 11- and 16-year-olds were simulated over a lifetime (up to 100 years of age) after vaccination. The same vaccine uptake rate was applied to all strategies. Costs included vaccination acquisition and administration, and IMD direct and indirect costs. Disutilities of IMD and LTS were applied to estimate losses of quality-adjusted life years (QALYs). Incremental cost-effectiveness ratio (ICER) was calculated as the difference in costs over QALY gained comparing a strategy of implementing the MenABCWY vaccine with the current standard of care (SoC). Extensive scenario analyses were conducted.

**Results:**

In the base case, compared with the SoC (one dose of a MenACWY vaccine at 11 years with a booster at 16 years, and two doses of a MenB vaccine at 16 years), introducing two doses of the MenABCWY vaccine at 11 years followed by a booster at 16 years (2+1 schedule) would prevent 63 IMD cases, 17 IMD cases with LTS, and 6 IMD-related deaths while providing savings (dominant ICERs). Results on ICERs, IMD cases, and IMD-related deaths were robust in scenario analyses when inputs most likely to influence the public health impact (i.e., IMD incidence and case fatality rate) were modified over plausible ranges.

**Conclusion:**

The MenABCWY vaccine fits the current vaccination platform. Results of the cost-effectiveness analysis demonstrate that the 2+1 schedule could offer the most public health benefits by providing the full protection against all five serogroups at an early age, while being a dominant economic strategy.

**Disclosures:**

**Katharina Schley, Dr.**, Pfizer Pharma GmbH: Employee|Pfizer Pharma GmbH: Employee|Pfizer Pharma GmbH: Stocks/Bonds|Pfizer Pharma GmbH: Stocks/Bonds **Jessica V. Presa, MD**, Pfizer Inc.: Stocks/Bonds|Pfizer Inc.: Stocks/Bonds **Vincenza Snow, MD**, Pfizer Vaccines: employee|Pfizer Vaccines: Stocks/Bonds **Alejandro D. Cane, MD, PhD**, Pfizer: Stocks/Bonds **Paula Peyrani, MD**, Pfizer, Inc: Employee|Pfizer, Inc: Stocks/Bonds **Ray Farkouh, PhD**, Pfizer: Stocks/Bonds **Shannon M. Sullivan, PhD**, Evidera: I am an employee of Evidera that is a consultancy to multiple pharma companies|Pfizer: Advisor/Consultant **Hossein Zivaripiran, PhD**, Pfizer: Advisor/Consultant|Pfizer: I am an employee of Evidera that is a consultancy to multiple pharma companies **Diana Teloian, MA**, Merck & Co., Inc: Grant/Research Support **Jörgen Möller, MSc**, Evidera: I am an employee of Evidera that is a consultancy to multiple pharma companies|Pfizer: Advisor/Consultant **J. Jaime Caro, MDCM, FRCPC, FACP**, Evidera: I am an employee of Evidera, a consultancy serving the pharmaceutical industry.|Pfizer: Advisor/Consultant

